# Apoptosis in virus infection dynamics models

**DOI:** 10.1080/17513758.2014.895433

**Published:** 2014-03-13

**Authors:** Ruili Fan, Yueping Dong, Gang Huang, Yasuhiro Takeuchi

**Affiliations:** ^a^ School of Mathematics and Physics, China University of Geosciences, Wuhan430074, China; ^b^ Graduate School of Science and Technology, Shizuoka University, Hamamatsu432-8561, Japan; ^c^ Department of Physics and Mathematics, Aoyama Gakuin University, Sagamihara252-5258, Japan

**Keywords:** apoptosis, virus dynamics, discrete delay, Hopf bifurcation, 92D30, 34K20

## Abstract

In this paper, on the basis of the simplified two-dimensional virus infection dynamics model, we propose two extended models that aim at incorporating the influence of activation-induced apoptosis which directly affects the population of uninfected cells. The theoretical analysis shows that increasing apoptosis plays a positive role in control of virus infection. However, after being included the third population of cytotoxic T lymphocytes immune response in HIV-infected patients, it shows that depending on intensity of the apoptosis of healthy cells, the apoptosis can either promote or comfort the long-term evolution of HIV infection. Further, the discrete-time delay of apoptosis is incorporated into the pervious model. Stability switching occurs as the time delay in apoptosis increases. Numerical simulations are performed to illustrate the theoretical results and display the different impacts of a delay in apoptosis.

## 1. Introduction

There are two different ways by which a cell can die: necrosis and apoptosis. Necrosis is a form of traumatic cell death that results from acute cellular injury, such as poison, a bodily injury, an infection or getting cut off from the blood supply. When cells die from necrosis, it is rather a messy affair. The death causes inflammation that can lead to further injury within the body. Apoptosis, or programmed cell death, on the other hand, is a naturally occurring process of cell suicide in response to a variety of physiological and non-physiological stimuli including chemical insults, virus infections and developmental cues, being essential in the maintenance of tissue development and homeostasis in the adult as well as in the regulation of immune responses [2,48]. The process of apoptosis shows a number of distinct characteristic morphological changes, including cell shrinkage and partial detachment from substratum, plasma membrane blebbing, chromatin condensation and intra-nucleosomal cleavage and ultimately cell fragmentation into apoptotic bodies which are phagocytosed without provoking an inflammatory response [37,50].

Apoptosis is genetically controlled and several viral gene products affect apoptosis by interacting directly with components of the highly conserved biochemical pathway which regulates cell death. Viruses may perform two functions. One is that viruses have evolved mechanisms to block the premature apoptosis of infected cells facilitating either the establishment and maintenance of persistent infection or prolonging the survival of lytically infected cells such that the production of progeny virus is maximized. The other function is that increasing number of viruses actively promote apoptosis, which is the culmination of a lytic infection and serving to spread virus progeny to neighbouring cells while evading the host inflammatory responses [37]. Viral induction of apoptosis occurs when one or several cells in a living organism is infected with a virus leading to cell death. Cell death in organisms is necessary for normal development of cells and cell cycle maturation. It is also critical in maintaining the regular functions and activities of cells.

Apoptosis is not only a key event in biological homeostasis but is also involved in the pathogenesis of many human diseases including acquired immune deficiency syndrome (AIDS) [1]. AIDS was first clinically observed from the US Centers for Disease Control in 1981, which is now defined as either a CD4+T-cell count below about 200 per microlitre blood, or the occurrence of specific diseases in association with certain HIV-related conditions and symptoms. Two years later the causative virus was identified by two separate research groups and afterwards named the human immunodeficiency virus (HIV) [29]. HIV is a retrovirus that primarily infects vital cells of the human immune system such as helper T-cells (specifically CD4+T-cells), macrophages and dendritic cells. The HIV infection usually leads to a progressive decay in the functionality and number of CD4+T-cells (normally about 1000 per microlitre in blood), resulting in a consequent impairment in host immune defenses and increasing susceptibility to opportunistic infections and malignancies for patients.

What causes the progressive depletion of the CD4+T helper cells leading to immunodeficiency in HIV infection?

The gradual decline of CD4+T-cells in HIV-infected patients is one of the most fundamental and controversial issues in AIDS research [21]. It has been reported that CD4+T-cell loss may be attributed to one of the following: (i) direct destruction by HIV cytopathic effects [33]; (ii) apoptosis induced by HIV proteins, such as Env, Tat (Tatanus antitoxin), Nef, Vpu and Vpr, which were proposed by Gougeon *et al.* in the 1990s [19,20] and reviewed by Gougeon [18]; (iii) excessive infection-induced immune cells activation drives CD4+T-cell depletion [14]; (iv) HIV-1-induced apoptosis in bystander uninfected cells [1]. Extensive body of studies has tried to address the phenomenon behind accelerated apoptosis in T-cells in HIV-infected patients since apoptosis has been suggested as another mechanism responsible for T-cell depletion since 1991 [1,3,5,7,14,17–22,33,38,48,57]. The role of bystander apoptosis induction in HIV infection and its role in disease progression is reviewed in [17]. Bystander apoptosis of neighbouring uninfected cells appears to encompass an explanation for most of the phenomenon observed during HIV infection that leads to progression to AIDS and remains one of the leading hypothesis for CD4+T-cell loss [17]. Analysis of blood cells derived from HIV-infected patients when cultured *in vitro* has revealed accelerated cell death infected as well as uninfected T-cells but, remarkably, the vast majority of the cells that undergo apoptosis are uninfected [47].

Nowadays, most researches are focused on the elucidation of apoptotic mechanisms. The possibility that modulating cell death by targeting specific factors involved in the whole process could be the key for cure of progression of HIV infection to AIDS, which is the also motivation of the work in this paper. Mathematical models can provide some insights into the dynamics of HIV viral load *in vivo* and may play a significant role in the development of a better understanding of HIV/AIDS and drug therapies. Here we mainly focus on qualitative understanding of the impact of apoptosis on virus infection dynamics by analysing several models with or without time delay.

The outline of the paper is as follows. In the next section, we incorporate the activation-induced apoptosis into virus infection model and analyse the impact of apoptosis of uninfected cells. In Section 3, we take into account specific cytotoxic T lymphocytes (CTLs) immune in HIV infection model including apoptosis. By analysing dynamical properties of the model, we give the potential impact of apoptosis. In Section 4, we focus on the existence of Hopf bifurcation when the apoptosis activation delay is present. Detailed numerical simulations are also given. The discussion of the mathematical results and of their biological implications are presented in Section 5.

## 2. Positive impact of apoptosis in viral dynamics

Mathematical models have been of central importance for understanding the dynamics of populations in an epidemiological context. Since the early 1990s numerous epidemic models have been used to describe the dynamics between viral infections and the immune response [6,8,25,26,36,39,44,46,51,53,55], particularly in the context of HIV infection [4,9,10,12,13,23,24,27,28,30,34,35,40–43,45,49,52,54,58]. The basic model for viral dynamics is given by the following simple three-dimensional system,

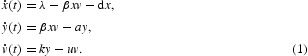

Here *x*(*t*) represents the concentration of uninfected or healthy cells at time *t, y*(*t*) represents the concentration of infected cells that produce virion at time *t* and *v*(*t*) represents the concentration of viruses at time *t*. λ is the rate at which new healthy cells are generated. *d* is the death rate of uninfected cells. β is the constant rate of infection of the healthy cells. *a* is the death rate of infected cells due either to the virus or to the immune system. Free viral particles are produced by infected cells at a rate *ky* and removed at a rate *uv*. All parameters 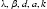 and *u* have positive values.

System (1) can be further simplified if we take into consideration that an average life span of viral particles is usually significantly shorter than one of infected cells. Therefore, it can be assumed that compared with a ‘slow’ variation of the infected cells level, the virus load *v*(*t*) relatively quickly reaches a quasi-equilibrium level. The equality 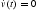 holds in the quasi-equilibrium state and hence 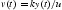. This assumption is referred to as ‘separation of time scales’ and is in common use in the virus dynamics [24,27,28,44]. We have to stress that this assumption does not imply that the virus concentration *v*(*t*) remains constant; on the contrary, it is assumed to be proportional to the varying concentration of infected cells *y*(*t*). Accordingly, system (1) can now be reformulated as a system of two ordinary differential equations,

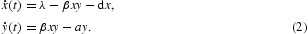

Virus infection and replication are often associated with apoptosis and this effect is likely to be responsible for much of the pathology associated with infectious disease. Many of the viruses associated with oncogenic transformation have adopted strategies for blocking apoptosis highlighting the centrality of this effect in carcinogenesis [37]. Indeed, these viral anti-apoptotic strategies can also contribute to the pathogenesis of virus infection and, in extreme situations, promote the oncogenic capacity of certain viruses. Some studies of the various mechanism used by viruses to suppress apoptosis have shed light on the fundamental biochemical pathways responsible for regulating programmed cell death [56]. Hence, understanding the mechanisms by which viruses regulate apoptosis may lead to the development of novel therapies for infectious disease.

Apoptosis is an important biological process that eliminates selected cells for the benefit of the whole organism. The ‘decision’ for apoptosis can come from the cell itself, or to be induced from its surrounding environment. We consider two different mechanisms by which the healthy cell commits suicide by apoptosis. One mechanism is generated by signals arising within the cell. Another is triggered by death activators binding to receptors at the infected cell surface. We use the following two models to describe those mechanisms.

### 2.1 Case I: apoptosis triggered by internal signals

One of the ways in which apoptosis occurs is by internal signals. When there is internal cellular damage, it triggers the release of a protein called BAX which punctures the mitochondrial membrane (Mitochondria handle cellular energy production). This causes Cytochrome C to leak from the mitochondria and bind with Apaf-1 (Apoptosis protease activating factor). The binding of Cytochrome C and Apaf-1 causes the formation of apoptosomes. This sets in motion a series of caspase formations which causes a structural breakdown in the cell and DNA destruction.

In this case, when apoptosis is taken into account in system (2), we have,

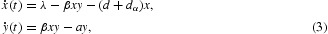

where *d* is the death rate due to necrosis, and *d*_α_ describes the death rate related to activation-induced apoptosis of uninfected cells.

The basic reproductive number of virus for system (3) is given by

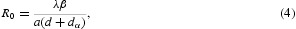

which describes the average number of infected cells generated from one infected cell at the beginning of the infection process. System (3) has two equilibria which are 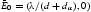 and 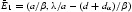 when *R*_0_>1.

The global dynamical properties of model (3) is clear, that is, all solutions of system (3) converge to the infection free steady state 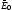 when *R*_0_≤1 and the infected steady state 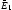 is globally asymptotically stable when *R*_0_>1. Obviously, the increasing of apoptosis parameter *d*_α_ could lead to the basic reproductive number *R*_0_ to less than 1, which means the virus load can be eliminated. In addition, there exists a threshold value *d*_0_, where 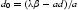, and when 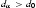, the level of infected cells tend to zero. From the term 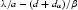, we know that increasing in *d*_α_ can decrease the load of infected cells. It shows that the apoptosis is useful to reduce the number of virus, which implies that apoptosis plays a positive role in preventing the spread of virus infection.

### 2.2 Case II: Apoptosis triggered by external signals

Another way that apoptosis can occur is through external signals. The previously mentioned protein called Fas on the cells’ surface can bind with another protein called tumour necrosis factor receptor. This binding will signal the cytoplasm and activate caspase 8 which in turn will set in motion a series of caspase formations. These series of caspase formations will ultimately cause phagocytosis (the cells being consumed by phagocytes). This process is often induced by T-cells which use the apoptosis process to destroy cells which are carrying viruses.

In the special case of lymphocytes, apoptosis plays an important role in optimizing the immune system by compensating lymphocytes proliferation through the elimination of cells that have become ill or ineffective. In HIV infection, apoptosis is a complex process that involves both HIV-infected and uninfected cells. HIV has evolved multiple mechanisms to promote survival for long enough to ensure a productive infection and this may be supported by the fact that infected cells do not undergo apoptosis as readily as uninfected bystander cells. This is also confirmed by direct evidence in lymph nodes where apoptosis was seen primarily in the uninfected bystander cells [15]. As reported in [38], lymph nodes of HIV-infected individuals contain a high percentage (with respect to uninfected individuals) of uninfected cells which are in an apoptotic state (that is which are ready to enter an apoptotic process).

As mentioned above, in HIV-infected persons, although both infected and uninfected cells undergo accelerated apoptosis, massive apoptosis was predominantly observed in uninfected CD4+T-cells present in lymph node, thymus or spleen. For the mathematical simplicity, we assume that all apoptosis-inducing factors of uninfected CD4+T-cells are directly correlated with the concentration of HIV-infected CD4+T-cells and since the chemical messengers inducing apoptosis are released by the infected CD4+T-cells, we further assume that the concentration of the chemical messengers inducing apoptosis is proportional to the concentration of the infected CD4+T-cells. Furthermore, when apoptosis of infected cells is not influenced by the presence of other cells, its effect is attributed to the death rate of infected cells. Consequently, it is more reasonable that the model (3) is modified as follows:

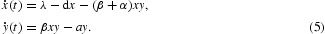

Here α is a positive parameter that represents the ‘apoptosis rate’ (also called apoptosis parameter) of uninfected cells in the presence of infected cells. It is reasonable to assume that α is bounded, that is, 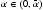. β is new per capita infection rate which describes both the probability of an infecting contact and the reproduction of virus. It should be pointed out that d *x* is a natural death rate of healthy CD4+T-cells. α *xy* is the death rate of healthy CD4+T-cells for the activation-induced apoptosis.

Consider system (5). There are two physically and biologically relevant non-negative equilibria, namely,

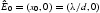
, infection or disease-free equilibrium, at which all individuals are susceptible and the population remain in the absence of disease.

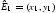
, positive or endemic equilibrium, where

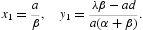

If 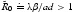, then *Ê*_1_ exists.

It is noteworthy that the basic reproduction number of infected cells *R˜*_0_ is independent of the apoptosis parameter α and the concentration of infected cells *y*_1_ of *Ê*_1_ is inversely proportional to the apoptosis parameter α.

### 2.3 Evolution of apoptosis parameter

**Figure 1.**
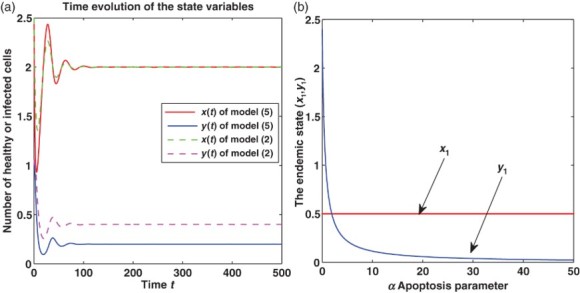
(a) The time evolution of the trajectory of system (2) and system (5) for healthy cells *x*(*t*) and infected cells *y*(*t*), respectively. The red line corresponds to *x*(*t*) and blue to *y*(*t*) in system (5), while the dashed green line corresponds to *x*(*t*) and the dashed magenta to *y*(*t*) in system (2). From (a), we can observe the number of infected cells of system (5) is less than that of system (2) in the endemic state. (b) The number of the healthy cells *x*_1_ and infected cells *y*_1_ in the endemic state of system (5) varying with the increasing of the apoptosis parameter α. From (b), we can observe that the healthy cells *x*_1_ does not vary with the increasing of α, however, the infected cells *y*_1_ decreases with the increasing of α.

We fix the parameter values as: 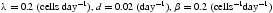, 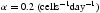, 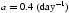. By calculation, we have 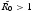, system (5) has an endemic steady state 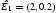 and *E*_1ˆ_ is globally asymptotically stable (Figure 1(a)). It can be expected that in the endemic state the healthy cells level *x*_1_ of system (5) is identical to that of system (2), but the infected cells level *y*_1_ of system (5) is lower than that of system (2). Furthermore, the infected cells level *y*_1_ reduces gradually with the increasing of apoptosis parameter α, which is illustrated by numerical simulation (Figure 1(b)).

The dynamical properties of system (5) shows that *R*_0˜_, which defines the average number of secondary infections generated by a typical infectious individual in a completely susceptible population in a steady demographic state, is a threshold parameter for the global stability of the infection-free equilibrium *Ê*_0_.

If 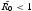, then on average an infected individual produces less than one new infected individual over the course of its infectious period, and the infection cannot be established among the individuals. Conversely, if 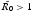, then each infected individual produces, on average, more than one new infection, and the disease can invade the population. Meanwhile, it is easy to see that in the case that 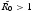, the levels of infected cell *y*_1_ inversely correlate with levels of apoptosis α. Hence, it is possible to speculate that apoptosis may contribute to eliminating cells which might prove harmful if they were to survive during viral infection. Indeed, as reported by O'Brien [37], by the method of apoptosis induction, viruses can induce host cell death while limiting inflammatory and other immune responses. Furthermore, promoting apoptosis may be used for treating diseases associated with a failure to trigger appropriate apoptosis [37].

## 3. Negative impact of apoptosis in HIV infection

It is commonly believed that the depletion of CD4+T-cells is the principal reason for the collapse of immune system. T-cell loss appears to be due to direct destruction by the virus or due to defective T-cell generation. Apoptosis has been suggested as another mechanism responsible for T-cell depletion during the progression of the HIV infection and an extensive body of recent literature is supporting this hypothesis [1,3,5,7,14,17–22,33,38,48,57]. When the level of CD4+T-cells drops from around 1000 cells per mm^3^ (the normal level for a healthy individual) to about 200 cells per mm^3^, the cell-mediated immunity is lost and the body becomes progressively more vulnerable to opportunistic infections. The T helper cells are unusual in the sense that they have no cytotoxic or phagocytic activity towards pathogens, and they do not kill infected cells or pathogens. Instead, T helper cells are involved in activating and directing other immune cells. It is noteworthy that activation of helper T-cells requires a much weaker activation stimulus than activation of cytotoxic T-cells. The role of CD4+T-cells in regulating and amplifying the immune response is vital, and a decline in their number results in deficits in humoral and cell-mediated immunity, opening an opportunity for opportunistic infections.

The Th immune response can be differentiated according to which type of the response is activated: Type 1 Th responses are critical in controlling intracellular infections via CTLs-mediated mechanisms (cell-mediated responses), whereas Type 2 helper responses are characterized by the activation of B-cells, which produce neutralizing (killing) antibodies (humoral immunity) [32]. The factors that determine which type of response will be activated are not fully understood [32]; however, in general, Th1 responses are more effective against intracellular pathogens (viruses, including HIV, and bacteria that inside host cells), while Th2 responses are more effective against extracellular bacteria, parasites and toxins. For this reason, in this paper we concentrate on the cell-mediated response, disregarding humoral immunity.

In order to describe the interactions between CD4+T-cells, HIV and CTLs response, it is usually assumed that the proliferation of immune response agents is proportional to their current concentration, the concentration of infected cells or a product (or a more complicated nonlinear function) of both (see [39,51] for comparison and discussion of models). An apparent deficiency of such a model is that it disregards the role of CD4+T-cells in immune response. This model deficiency is acceptable for the majority of viral infections, where the CD4+T-cell level remains approximately constant throughout the course of the infection. For HIV infection, however, CD4+T-cells are the target cells, and a decrease in their levels affects the efficacy of immune system. The experimental findings by Borrow *et al.* [11] show that CD40 ligand-mediated, which is a glycoprotein that is transiently expressed at high levels on the surface of CD4+T-cells when they are activated, interactions are involved in the generation of CTLs. In accordance with experimental findings and to overcome the deficiency of disregarding the role of CD4+T-cells in immune response, in this paper we assume that the establishment of a lasting CTLs response depends on CD4+T-cell help, and that HIV impairs T helper cell function. Thus, the activation and proliferation of CTLs depend on three concentrations, namely on the current concentration of CTLs, the concentration of infected cells (antigen-presenting cells) and the concentration of the healthy CD4+T-cells. This assumption was firstly introduced by Wodarz and Nowak [52]. Afterwards, Huang *et al.* [24] discussed a possible mechanism which eventually enables HIV to break from immune control by the model with this assumption via continuous mutations and evolution.

Based on the above assumption, we consider the following model with CTLs immune responses:

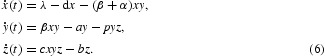

Here, *x*(*t*), *y*(*t*) and *z*(*t*) are the concentration of healthy CD4+T-cells, the infected cells, antigen-specific CTLs, respectively. The infected cells are killed by CTLs at a rate *pyz*. The specific CTLs proliferates at a rate *cxyz* (which is proportional to the levels of healthy CD4+T helper cells, antigen-presenting cells and CTLs) and decays at a rate *bz*.

It is noteworthy that in the form *xyz*, the multiplier *y*(*t*) is responsible for antigen-presenting, describing the level of antigen-presenting cells, which is assumed to be proportional to the infected cells level. The assumption that the concentration of antigen-presenting cells is proportional to the infected cells concentration is fairly common in mathematical immunology.

There are three physically and biologically relevant types of non-negative equilibria, namely

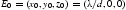
, infection-free or disease-free equilibrium, at which all individuals are susceptible and the population remains in the absence of disease. *E*_0_ always exists.

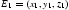
, immune-absence equilibrium, where

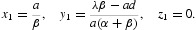

If 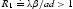, then *E*_1_ is nonnegative and therefore biologically meaningful.

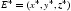
, interior immune-presence equilibrium, where

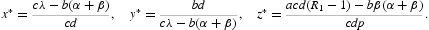

If 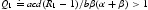
(and hence 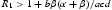), or 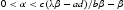, then E* is nonnegative and therefore biologically meaningful.

Here, *R*_1_ is called the basic reproduction number of infected cells of system (6) (that is, *R*_1_ is an average number of infected cells produced by a single infected cell introduced into entirely healthy environment), and *Q*_1_ is called the basic reproduction number of immune response of system (6) (that is, *Q*_1_ is an average number of CTLs produced by a single CTLs introduced into a system where healthy and infected cells are at their equilibrium levels). Therefore, depending on values of *R*_1_ and *Q*_1_(α), system (6) has three equilibria.

**Figure 2.**
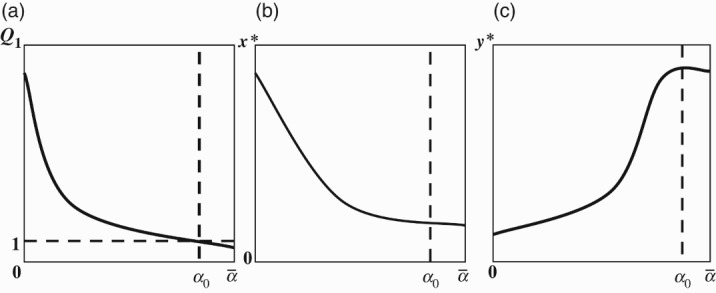
(a), (b) and (c), respectively, show the basic reproduction number of immune response *Q*_1_ with respect to α, the number of healthy cells x* and the number of infected cells y* in the immune-presence equilibrium with respect to α. From (a), we can derive *Q*_1_=1 at α=α_0_, *Q*_1_>1 when 0<α<α_0_ and *Q*_1_<1 when α_0_<α<α¯. (b) and (c) show the number of healthy cells decreases to a stable value with the increasing of α satisfying α_0_<α<α¯, while that of infected cells increases.

The basic reproduction number of immune response *Q*_1_ decreases with increasing α. Furthermore, there is α_0_ (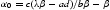) such that 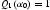; *Q*_1_>1 for all 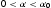, and *Q*_1_<1 for all 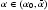 (Figure 2(a)).

When 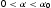, namely *Q*_1_>1, the immune presence equilibrium E* exists. In this case, the concentration of infected cells y* grows with increasing α, whereas the concentration of healthy cells decreases with increasing α (see Figure 2(b) and (c)). This interval of α might correspond to the asymptomatic stage of HIV infection, where the viral load gradually grows and the healthy cells decrease. Hence the apoptosis further accelerates the progression of the HIV infection to AIDS in the ranges 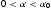. When 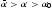, which means *Q*_1_<1 and *R*_1_>1, the immune absence equilibrium *E*_1_ exists and the concentration of viruses decreases with the increasing of α for 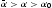.

### 3.1 Stability analysis of three equilibria states

To study the local stability of the steady states of model (6), we linearize the system at 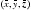 and obtain the following Jacobian matrix:

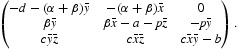

(i) The characteristic equation of system (6) at *E*_0_ is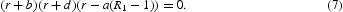  Roots of this equation are *r*_1_=−*b, r*_2_=−*d* and 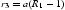. Hence, *R*_1_<1 is sufficient to ensure the asymptotic stability of *E*_0_. When *R*_1_>1, Equation (7) has one positive real root, therefore *E*_0_ is unstable. When *R*_1_=1, Equation (7) has a zero solution.
(ii) The characteristic equation of system (6) at *E*_1_ is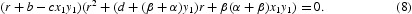  Here root 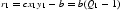 is negative when *Q*_1_<1, positive when *Q*_1_>1, and zero when *Q*_1_=1. Furthermore, when *E*_1_ exists, all coefficients of quadratic equation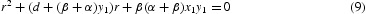  are positive, and hence both roots of Equation (9) have negative real parts. That is, *Q*_1_<1 ensures that all eigenvalues of Equation (8) have negative real parts. Hence the immune-absence equilibrium state *E*_1_ is locally asymptotically stable if *R*_1_>1 and *Q*_1_<1, and is unstable if *Q*_1_>1. When *Q*_1_=1, it is a critical case.
(iii) The characteristic equation of system (6) at E* is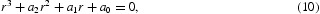  where

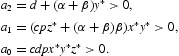

Since

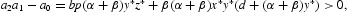

by the Routh–Hurwitz theorem, all roots of (10) have negative real parts, and the immune-presence equilibrium state E* is locally asymptotically stable if *Q*_1_>1.

Theorem 3.1 Consider system (6).

(i) The infection-free equilibrium state *E*_0_ is locally asymptotically stable if *R*_1_<1, is unstable if *R*_1_>1 and if *R*_1_=1, it is a critical case.
(ii) The immune-absence equilibrium state *E*_1_ is locally asymptotically stable if *R*_1_>1 and *Q*_1_<1, is unstable if *Q*_1_>1 and if *Q*_1_=1, it is a critical case.
(iii) The immune-presence equilibrium state E* is locally asymptotically stable if *Q*_1_>1.

### 3.2 Numerical simulations and biological meanings

It is shown in Theorem 3.1 that *R*_1_ is a threshold parameter for the local stability of the infection-free equilibrium *E*_0_. For 0<*R*_1_<1, the infection-free equilibrium state *E*_0_ is unique equilibrium state of the system (6) and is locally asymptotically stable (it can be conjectured that it is the global attractor of the system: phase trajectories with non-negative initial conditions eventually converge to this equilibrium state). The concentration of healthy CD4+T-cells reaches its maximum possible level λ/*d* in this equilibrium state. When 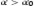, the model (6) has two equilibrium states: in addition to the infection-free equilibrium state *E*_0_, an immune-absence equilibrium state *E*_1_ appears. For these ranges of α, the infection-free equilibrium loses its stability and turns into a saddle point, whereas the immune-absence equilibrium *E*_1_ is asymptotically stable. The immune-absence equilibrium state corresponds to a situation where both healthy and infected CD4+T-cells are present, while the antigen-specific CTLs response is not activated. When 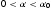 (and hence *Q*_1_>1 holds), the system (6) has three equilibria: in addition to the infection-free equilibrium state *E*_0_ and the immune-absence equilibrium state *E*_1_, an asymptotically stable immune-presence equilibrium state E* where all three components of system are present appears in the positive quadrant. Here we define α_0_ as the immunodeficiency threshold, which is a threshold parameter for the stability of the immune-presence equilibrium E*.

Now we fix the parameter values as

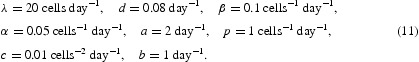

**Figure 3.**
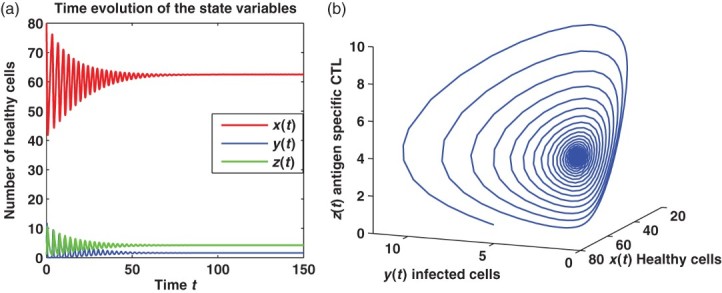
(a) and (b) The time evolution of the trajectory of system (6) for healthy cells *x*(*t*), infected cells *y*(*t*) and antigen-specific CTLs *z*(*t*). In (a), the red line corresponds to *x*(*t*), blue line corresponds to *y*(*t*) and green line corresponds to *z*(*t*). The panel (b) is the phase plane of system (6). They show that the immune-presence equilibrium state E* is locally asymptotically stable for *R*_1_>1 and *Q*_1_>1.

By calculation, we have *R*_1_=12.5 and *Q*_1_=1.23. There exists the immune-presence equilibrium state 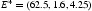 and E* is asymptotically stable (see Figure 3).

It is well known that, during the second phase (the chronic or asymptomatic phase) of HIV infection, the virus within-host diversity increases and the number of host CD4+T-cells decreases because they are the primary target of virus. Further, the third phase or AIDS phase is characterized by a dramatic loss in CD4+T-cells and a strong increase of viral load. Clinically, the onset of AIDS is defined as the time point at which the CD4+T-cells count in the blood falls below 200 per microlitre. In this paper, the characteristics of the chronic phase and AIDS phase are obviously displayed with the variation of the apoptosis parameter α and the activation-induced apoptosis may promote HIV infection procession, that is, the effect of activation-induced apoptosis may be detrimental to the immune system. As we mentioned above, α_0_ is the immunodeficiency threshold. When 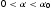, that is, *Q*_1_>1, all solutions of system (6) converge to the immune-presence equilibrium state E*, and the number of infected CD4+T-cells increases with increasing α, whereas the number of the healthy CD4+T-cells and the number of specific CTLs decrease with increasing α.

At 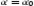, that is *Q*_1_=1, the asymptotically stable immune-presence equilibrium state E* disappears. When 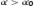, solutions of system (6) converge to the immune-absence equilibrium state *E*_1_, which is asymptotically stable. Furthermore, as α increases further, the number of infected cells gradually decreases. In terms of theory, it seems to be that when 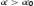, that is, the immune system has not been activated, the apoptosis may benefit to the infected host. In fact, many viruses have adopted strategies for blocking apoptosis [37,56]. Hence, the level of infected cells may not be decreasing.

## 4. Effects of time delay in apoptosis

Due to the complexity of delay differential equations, many scientists do not include delays in their models. However, many biological processes have inherent delays and models including them may lead to additional insights into the study of complicated biological processes. Earlier work in modelling dynamics between viruses and immune responses was presented by Nowak and Bangham [36]. Tam [46] incorporated a discrete delay into one of Nowak and Bangham's models. Recently, Zhu and Zou [58] considered the dynamical properties of an HIV-1 infection model with CTLs immune response and an intracellular delay.

In HIV-infected persons, both infected and uninfected CD4+T-cells undergo accelerated apoptosis *in vitro* and *in vivo*. Apoptosis occurs mainly in bystander uninfected cells, whereas productively HIV-infected cells have evolved strategies to prevent or delay apoptosis in the context of immune activation [3]. Furthermore, it was determined that intracellular expression of HIV-1 Tat was able to delay Fas-mediated apoptosis and this effect was due to the presence of the second exon which is the protein, and causes a persistent infection in the host [31]. It is speculated that time delay of the apoptosis of uninfected CD4+T-cells may play an important part in the progression of HIV infection. Thus, we incorporate time delay of apoptosis into HIV infection model (6) where the delay describes the latent period from the contacted cells with viruses to activating apoptosis and obtain the following model:

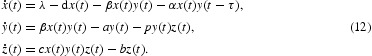

Here τ is called the intracellular delay describing the phase in which target cells are infected until uninfected cells start apoptosis in presence of infected cells.

Systems (12) and (6) have the same equilibrium states. The basic infection reproductive number and the basic reproduction number of immune response of system (12) are also, respectively,

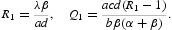



Next, we discuss the local stability of equilibrium states and the existence of Hopf bifurcations of system (12) when τ>0.

### 4.1 Positivity and boundedness

We denote by *C* the Banach space of continuous functions 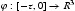 equipped with the sup-norm

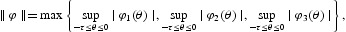

where 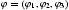. Further, let

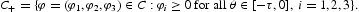



The initial condition of system (12) is given as

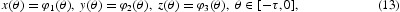

where 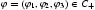.

The following proposition establishes the non-negativity and boundedness of the solutions of (12) with (13).

Proposition 4.1 Let 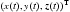 be any solution of system (12). Then under the initial conditions (13), all solutions 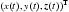 are non-negative on [0,+∞) and ultimately bounded.

*Proof* If *x*(*t*) were to lose its non-negativity on some local existence interval [0, *T*) for some constant *T*>0, there would be the first time at *t*_1_>0 such that *x*(*t*_1_)=0. By the first equation of (12) we have 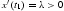. That means *x*(*t*)<0 for 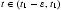, where ϵ is an arbitrarily small positive constant. This leads to a contradiction. It follows that *x*(*t*) is always positive. Further, from the second and the third equations in (12), we have, respectively,

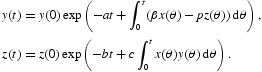

Then, it is easy to see that *y*(*t*) and *z*(*t*) are non-negative on [0, *T*).

For *t*∈[0, *T*), we have from (12) that 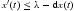. The well-known comparison principle implies that *x*(*t*) is bounded on [0, *T*), i.e. 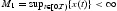. We again have from Equation (12) that on [0, *T*),

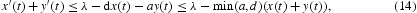

which implies there exists 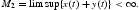 Since 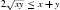, it has

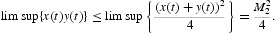

Then by

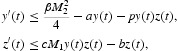

we have

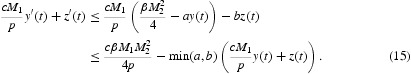

Thus there exists 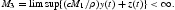 Therefore by comparison principle, *y*(*t*) and *z*(*t*) are also bounded on [0, *T*). Boundedness of the solution 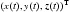 implies that the local existence interval [0, *T*) can be continued to *T*=+∞. This proves that the solution 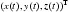 exists and is non-negative on [0,+∞). The inequality (14) implies that *x*(*t*)+*y*(*t*) is ultimately bounded, and so are *x*(*t*), *y*(*t*). By the inequality (15), *z*(*t*) is also ultimately bounded. This completes the proof.

To study the local stability of the steady states of model (12), we linearize the system at 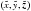 and obtain the following characteristic equation:

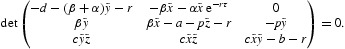



### 4.2 Stability analysis of the infection-free equilibrium

The characteristic equation of system (12) at the infection-free equilibrium *E*_0_ is

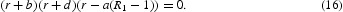

Roots of Equation (16) are *r*_1_=−*b, r*_2_=−*d* and 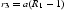, hence *R*_1_<1 is sufficient to ensure the asymptotic stability of *E*_0_. When *R*_1_>1, Equation (16) has one positive real root, thus *E*_0_ is unstable. When *R*_1_=1, Equation (16) has a zero solution. Therefore, we have the following theorem.

Theorem 4.2 Consider system (12).

(i) If *R*_1_<1, then the infection-free equilibrium *E*_0_ is locally asymptotically stable for any time delay τ>0, and the disease cannot invade the population.
(ii) If *R*_1_>1, then the infection-free equilibrium *E*_0_ is unstable for any time delay τ>0, and invasion is always possible.

### 4.3 Stability analysis of the immune-absence equilibrium

The characteristic equation of system (12) at the immune-absence equilibrium *E*_1_ is

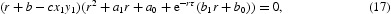

where

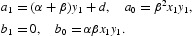



Here the root 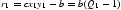 is negative when *Q*_1_<1, positive when *Q*_1_>1 and zero when *Q*_1_=1. Now, we consider the equation

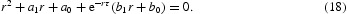

Since *r*=0 is not a root of Equation (18) for any τ>0 and all roots have negative real parts for τ=0, when *Q*_1_<1, as the delay τ increases, the root of Equation (18) can only enter the right-half plane in the complex plane by crossing the imaginary axis except the origin. If 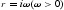 is a purely imaginary root of Equation (18), then

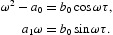

From which, we have that

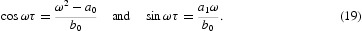

Noting that 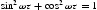, it follows that

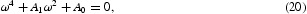

where

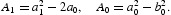



Letting *s*=ω^2^ gives

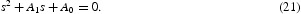

Denote

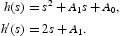

Equation (21) has at least one positive real root in two circumstances.

- If 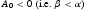, since the leading coefficient is positive, then there is a positive real root.
- If 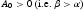, the roots of Equation (21) are

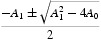

and there are two simple positive roots if and only if *A*_1_<0 and 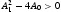.

Without loss of generality, we assume that Equation (21) has two positive roots denoted by *s*_1_ and *s*_2_, respectively. Then Equation (20) has two positive roots 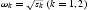. From Equation (19), we have

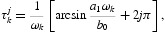

where *k*=1, 2; *j*=0, 1, … . Then 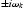 is a pair of purely imaginary roots of Equation (20) with 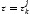.

Define

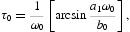

where

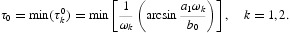

Denote 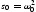, which is a positive root of Equation (21).

Assume

(*H*_1_) *A*_0_<0 or *A*_0_>0, *A*_1_<0 and 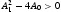;
(*H*_2_) *h*′(*s*_0_)>0.

By implicit differentiation of Equation (18) with respect to τ, we obtain

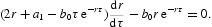

Hence

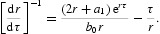

If the hypothesis (*H*_2_) is satisfied and noting Equation (19), we have

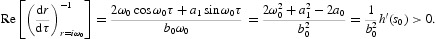

Then

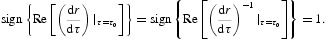

Therefore, for delay τ<τ_0_, roots of Equation (18) have negative real parts. When τ=τ_0_, there exist a pair of purely imaginary roots for Equation (18). When τ>τ_0_, Equation (18) has at least one positive real root.

From the above analysis, we have the following theorem.

Theorem 4.3 Consider system (12), when *R*_1_>1, *Q*_1_<1 and the conditions (*H*_1_) and (*H*_2_) hold,

(i) if 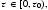 then the immune-absence equilibrium state *E*_1_ is locally asymptotically stable;
(ii) if 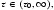 then the immune-absence equilibrium state *E*_1_ is unstable and system (12) undergoes a Hopf bifurcation at *E*_1_ when τ=τ_0_.

### 4.4 Stability analysis of the immune-presence equilibrium

The characteristic equation of system (12) at the immune-presence equilibrium E* is

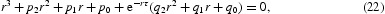

where

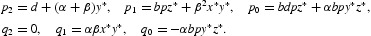

It is found from Theorem 3.1 that when τ=0, the roots of Equation (22) have negative real roots. By the continuous dependence of roots of Equation (22) on the parameters, it follows that there exists a τ_0_>0 such that for 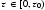, all the roots of Equation (22) will satisfy Re(*r*)<0 and when τ=τ_0_, Re(*r*)=0.

To determine this τ_0_, we assume 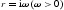 is a solution of Equation (22) and separate real and imaginary parts, and obtain

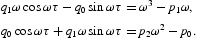

From which, we have that

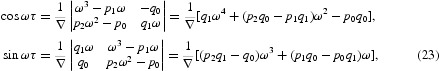

where

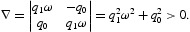

Noting that 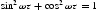, it follows that

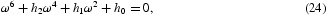

where

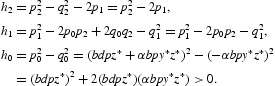



Letting *s*=ω^2^ gives

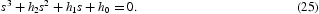

Denote

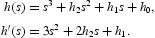



We utilized the Sturm chain which is proposed in [16] to extend the sufficient conditions to preserve that the polynomial (25) has at least one positive real root. Since *h*_0_>0 and the polynomial (25) is of odd degree, we are guaranteed that Equation (25) has a negative real root. From Theorem 1 of [16], we obtain the sufficient conditions to ensure one positive real root for the polynomial (25). That is,

- *h*_0_, *h*_1_ and *h*_2_ are not all positive;

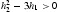
;

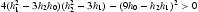
.

The above conditions can also ensure that Equation (25) has two positive real roots. We denote them by *s˜*_1_ and *s˜*_2_, respectively. Then Equation (24) has two positive roots 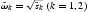. From Equation (23), we have

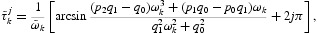

where *k*=1, 2; *j*=0, 1, … . Then 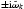 is a pair of purely imaginary roots of Equation (24) with 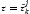.

Define

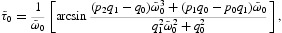

where

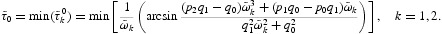



Denote 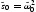, which is a positive root of Equation (25).

Assume

(*H*_3_) *h*_0_, *h*_1_ and *h*_2_ are not all positive;
(*H*_4_) 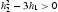; 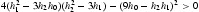;
(*H*_5_) 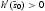.

By implicit differentiation of Equation (22) with respect to τ, we obtain

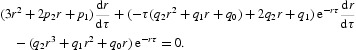

Hence, *q*_2_=0

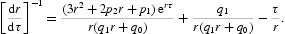



If the hypothesis (*H*_3_) is satisfied and noting Equation (23), we have

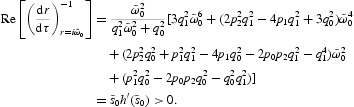

Then

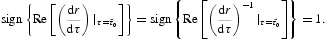

Therefore, when the delay 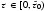, the roots of Equation (22) have negative real parts. When 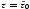, there exist a pair of purely imaginary roots for Equation (22), and other roots have no negative real parts.

From the above analysis, we have the following theorem:

Theorem 4.4 Consider system (12), when *Q*_1_>1 and conditions (*H*_3_)–(*H*_5_) hold, we have

(i) if 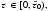 then the immune-presence equilibrium E* is asymptotically stable;
(ii) if 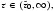 then the immune-presence equilibrium E* is unstable and system (12) undergoes a Hopf bifurcation at E* when 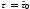.

### 4.5 Numerical simulations

By using suitable numerical methods, we demonstrate that stability change would occur at the branch of the equilibria *E*_1_ and E* of system (12), leading to Hopf bifurcations.

First, we fix the parameter values as

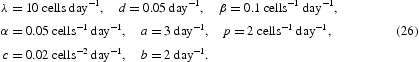

**Figure 4.**
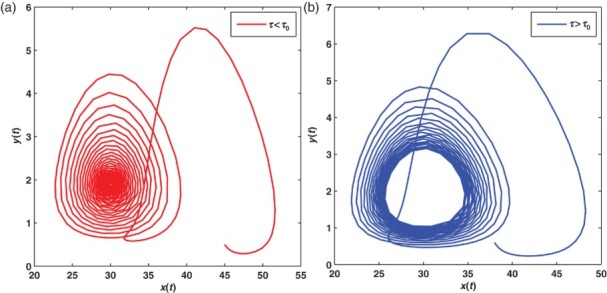
The diagrams show the time evolution of trajectory of system (12) including the time delay of apoptosis for healthy cells *x*(*t*), infected cells *y*(*t*) and antigen-specific CTLs *z*(*t*) for *R*_1_>1 and *Q*_1_<1. In (a), it is shown that when τ<τ_0_, the solution of Equation (12) tends to the immune-absence equilibrium state *E*_1_ for the given initial value. In (b), it is shown that when τ>τ_0_, the solution of Equation (12) no longer tends to the equilibrium state *E*_1_. Instead, a periodic solution appears.

By direct calculation, we have *R*_1_=6.67, *Q*_1_=0.57, *A*_1_=−1.0222, *A*_0_=0.2408, 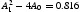 and 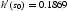. Note that the conditions *H*_1_ and *H*_2_ hold. We obtain 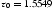 by calculation, and the simulation displays *E*_1_=(30, 1.8889, 0) is asymptotically stable when τ=1.4 (Figure 4(a)), while it loses its stability and a periodic solution emerges when τ=1.6 (Figure 4(b)).

**Figure 5.**
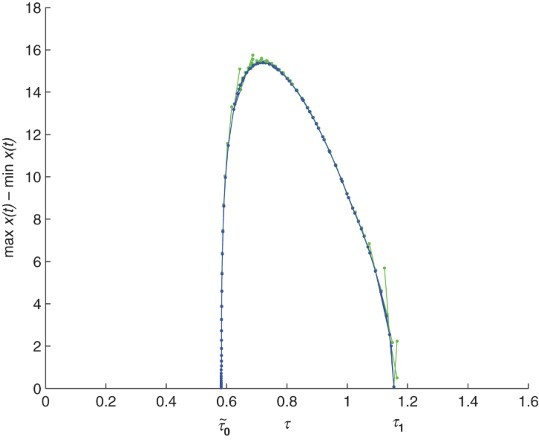
Bifurcation diagram of system (12) with parameter values given in (11).

**Figure 6.**
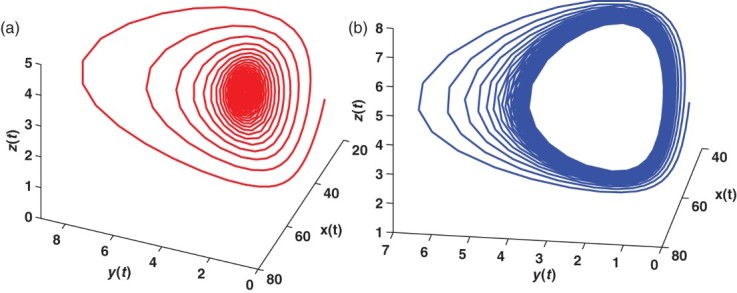
(a) and (b) The time evolution of trajectory of system (12) including the time delay of apoptosis for healthy cells *x*(*t*), infected cells *y*(*t*) and antigen-specific CTLs *z*(*t*), for *R*_1_>1 and *Q*_1_>1. In (a), it is shown that when τ<τ˜_0_, the solution of system (12) tends to the equilibrium E* for the given initial value. In (b), it is shown that when τ>τ˜_0_, the solution of Equation (12) no longer tends to the equilibrium E*. Instead, a periodic solution appears.

Next we choose the parameter values given by (11). We have *R*_1_=12.5, *Q*_1_=1.23 and three equilibrium states exist. In this case, we have 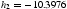, *h*_1_=26.8773, *h*_0_=0.3468, 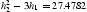 and 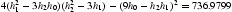, which make the conditions (*H*_3_−*H*_5_) hold. By Theorem 4.4, the first Hopf bifurcation occur at 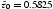. We use a Matlab package called DDE-BIFTOOL to draw the graph of Hopf branches in Figure 5. We use a Matlab package DDE23 to find numerical solution to system (12) with τ=0.45 and 0.6. As shown in Figure 6 we observe that 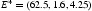 is asymptotically stable when τ=0.45, while it loses its stability and a periodic solution emerges when τ=0.6. Comparing Figure 4 with Figure 3 we see that increasing the time delay brings rich dynamical behaviour.

## 5. Discussion and conclusion

As a result of HIV infection, CD4+T-cells loss may be due to direct viral killing infected cells or killing infected CD4+T-cells by CTLs. Recently apoptosis has been suggested to be another mechanism responsible for CD4+T-cells depletion. In this paper, we propose several models which include the activation-induced apoptosis phenomenon and analyse the influence of apoptosis on viral infection dynamics, especially HIV infection dynamics.

In model (3), we use the constant *d*_α_ to describe the death rate of uninfected cells related to activation-induced apoptosis. The analysis for system (3) shows that there exists a threshold *d*_0_ when *R*_0_=1. When *d*_α_ is below this threshold, virus is able to invade and persist within a host. Once *d*_α_ becomes more than this threshold, the level of infected cells gradually decreases with increasing of *d*_α_ and finally infected cells tend to vanish. Further, we postulate that the concentration of chemical messengers released by infected cells and inducing apoptosis is proportional to that of the infected cells and obtain model (5). The analysis for system (5) illustrates that when the basic reproduction number of virus is more than 1, the endemic equilibrium is stable and the number of infected cells at this equilibrium decreases with the increasing of apoptosis parameter. These theoretical analyses for models (3) and (5) illustrate that an increase in apoptosis can bring about a fall in the number of infected cells, which implies that apoptosis plays a positive role in preventing virus infection.

In HIV infection, immune response has been shown to be universal and necessary to eliminate or control the disease. To recover from a viral infection, the CTLs will clear away the infected cells to prevent further viral replications. Since CTLs play a critical role in antiviral defense by attacking cells infected with HIV, we consider the influence of apoptosis in HIV infection model with the immune response. Here, we assume that the activation of proliferation of CTLs depends on the concentration of CTLs, the concentration of infected CD4+T-cells and the concentration of healthy CD4+T-cells, and we obtain model (6). According to the qualitative analysis for model (6), we find that there is a threshold, namely the immunodeficiency threshold α_0_, which is specific for HIV dynamics. When the apoptosis parameter is below this threshold, the immune system is effective in virus control, and all solutions of system (6) converge to the immune-presence equilibrium in which the number of infected CD4+T-cells increases with rise of the apoptosis parameter (although the concentration of healthy CD4+T-cells and CTLs decrease). Furthermore, once the apoptosis parameter becomes beyond this threshold, the immune response vanishes and the level of infected cell decreases with the raise of apoptosis parameter. From the above results, we find that the apoptosis phenomenon may accelerate the depletion of CD4+T-cells and destroy the immune response for the ranges 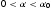, whereas for 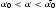, the apoptosis may boost the rate of decline in infected cells. Therefore, the activation-induced apoptosis may aggravate or comfort the HIV infection for some ranges of apoptosis parameter.

In reality, during HIV infection, the intracellular expression of full-length Tat is able to delay Fas-mediated apoptosis, hence there exists a time lag from viruses contacted to activating apoptosis. Hence, we incorporate time delay of apoptosis into model (6). We study the impact of the time delay in apoptosis by analysing model (12). By using a combination of bifurcation theory and numerical simulations, we find that there exists the limit cycle with increased time delay. By associated transcendental characteristic equations for the immune-free equilibrium of system (12) with the delay, we show that there exists the critical value denoted by τ_0_. When τ is less than this critical value, the infection-absence equilibrium of system (12) is locally asymptotically stable; when τ is more than τ_0_, this equilibrium is unstable and a Hopf bifurcation occurs at τ=τ_0_. When *Q*_1_ is larger than 1, we obtain results for the immune-present equilibrium similar to those we obtained for the immune-free equilibrium. Therefore, the time delay in apoptosis could lead to very complicated dynamics including stable periodic solution, which gives additional insights into the study of the apoptosis effect on the HIV infection.

The principal conclusion in this paper is that apoptosis is helpful for inhibition of the virus level by cells itself, while to some extent it is dangerous for HIV infection as it may cause fall in CD4+T-cells which could lead to collapse of CTLs immune system. Furthermore apoptosis delay can give rise to a viral oscillation in the host.
